# Temporal proteome profiling of *Botrytis cinerea* reveals proteins involved in plant invasion and survival

**DOI:** 10.1038/s41598-025-92683-5

**Published:** 2025-04-07

**Authors:** Shriya Singh, Manasa Hegde, Inderjeet Kaur, Nidhi Adlakha

**Affiliations:** 1 Synthetic Biology and Bioprocessing Group, Regional Centre for Biotechnology, NCR-Biotech Cluster, Faridabad, Haryana India; 2 Department of Biotechnology, Central University of Haryana, Mahendergarh, Haryana India

**Keywords:** *Botrytis cinerea*, Optimized media, Phytopathogen, Proteomics, Temporal protein dynamics, Virulence factors, Fungi, Proteomics

## Abstract

*Botrytis cinerea* is a necrotrophic fungal pathogen that poses a significant threat to many crops. Understanding the proteome dynamics of phytopathogens during infection can help combat plant diseases. However, most proteomics studies in phytopathogens face interference from abundant host proteins. Here, we optimized a solid media that better mimics *in-planta* conditions and used it to perform the temporal protein dynamics in *Botrytis cinerea*. An agar media with 20% tomato fruit extract and 2% deproteinised leaf extract was utilized for label-free quantitative proteomics at 12, 36, 72 and 120 hpi. Out of 3244 quantified proteins, 2045 showed differential regulation. Glycosyl hydrolases, pectin esterases, stress protein DDR48, RhoGEF and essential transcription factors were found to be upregulated during the early phase, highlighting their role in fungal virulence. Meanwhile, pathways such as macromolecule synthesis, purine, and carbohydrate metabolism were upregulated in the late-growth phase. Overall, the study provides a comprehensive understanding of proteome dynamics during Botrytis infection.

**Supplementary Information:**

The online version contains supplementary material available at 10.1038/s41598-025-92683-5.

## Introduction

*Botrytis cinerea *is a necrotrophic plant pathogen responsible for gray mold disease. It affects a wide range of crops globally and causes severe economic losses^1,2^. Like other necrotrophs, they acquire nutrients for survival by colonising plants and secreting large amounts of virulence factors^3,4^.This opportunistic fungus infects plants growing in the field and spreads in post-harvest storage^5,6^. Hence, this fungus alone results in an annual global economic loss of about US$100 billion.

Researchers have studied how *Botrytis *sp. invades plants and causes disease to discover ways to prevent widespread damage. This includes examining the molecular interactions between the fungus and the plant, along with the environmental factors that may influence the severity of the disease. *Botrytis* sp. secretes a range of enzymes including pectin lyase^7,8^, pectin methylesterase^9–11^, exo and endo polygalacturonase^12,13^, cellulases^14^, and proteases^15,16^, which aid in breaking down complex cell walls, enabling the fungus to establish itself in plants. For instance, deletion of endopolygalatouranase, along with protease and lipase, significantly reduced lesion size, highlighting their role in the intercellular proliferation of invading Botrytis hyphae^17^. This indicates that the fungus’s ability to cause disease likely depends on multiple proteins rather than a single enzyme, highlighting the importance of comprehensive proteomics research.

Identification of fungal proteins involved in mediating infection in plants is crucial in combating the disease^18,19^. Proteomic approaches have been used earlier to analyse the apoplast during pathogen infection, which led to the identification of key players involved in plant-pathogen interactions^20^. Disruption of these factors has been shown to eliminate the virulence and cause irregularities in the formation of infection cushions^21,22^. This suggests the effectiveness of proteomics as a tool for identifying virulence factors in phytopathogenic fungi. However, understanding the molecular events in *Botrytis* sp. during the course of infection remains challenging due to studies being limited to either the early or late phase of invasion. This limitation is primarily due to inherent difficulties in extracting proteins from phytopathogens as the abundant plant proteases degrade fungal proteins during protein extraction.

Additionally, plant cell wall components, the extracellular matrix, and other plant-derived compounds hinder the efficient extraction of fungal proteins. The abundant plant background such as Rubisco (ribulose bisphosphate carboxylase oxygenase) masks the less dominant fungal proteins limiting mass spectrometry based proteomic analysis^23^. Therefore, a simplified model where the proteome of the plant and its associated fungi can be easily separated without affecting their molecular interplay is considered an appealing alternative.

Many researchers have used host tissue extracts instead of the host itself to understand the influence of host plants on fungal proteome. Plant pathogens have been shown to respond effectively to the presence of leaf extracts from susceptible host plants (sweet orange), releasing multiple virulence factors^24^. Similarly, another group examined the induction of proteome in phytopathogen by passion fruit leaf extract^25^. This indicates that an optimized media that closely mimics *in planta* conditions can provide an attractive alternative for studying host-mediated fungal proteome dynamics.

Therefore, we have designed a rational study to develop solid support for proteomics research in *Botrytis cinerea*. Using biochemical and proteomic approaches, we have validated the solid support media equivalent to the *in planta* condition. Further, a stage-specific quantitative proteomics study was performed in *B. cinerea*, which allowed us to identify proteins and effectors responsible for initiating the invasion process in fungi. We have also identified proteins required to maintain invasion and survival in phytopathogenic fungi during the late phase of infection.

## Results and discussion

### Comparative evaluation of plant pathogenicity

Analysing protein dynamics in phytopathogenic fungi is seen as an effective method for identifying targets for theragnostic development. However, the presence of plant host proteins in fungal samples complicates the identification of stage-specific virulence proteins. Therefore, it is essential to identify media conditions that closely mimic those of the plant host. This will aid in conducting proteomics experiments in fungi by minimizing the interference caused by an abundant host background.

For our study, we have considered *Botrytis *sp., a necrotrophic fungi whose proteome has shown a preponderance of pathogenic enzymes that mediate plant infection^26^. However, different species of Botrytis vary in host plants, pathogenicity, and other relevant properties affecting disease control. Previous study has also shown differential expression of proteins in six isolates of *Botrytis cinerea*^27^. Therefore, we procured Botrytis strains from various culture collection centres and evaluated them for their ability to cause plant diseases. The screening for virulence trait in *Botrytis* sp. was performed based on multi-dimension analysis, including detached leaf assay, fruit inoculation and biochemical assays.

**Fig. 1:**
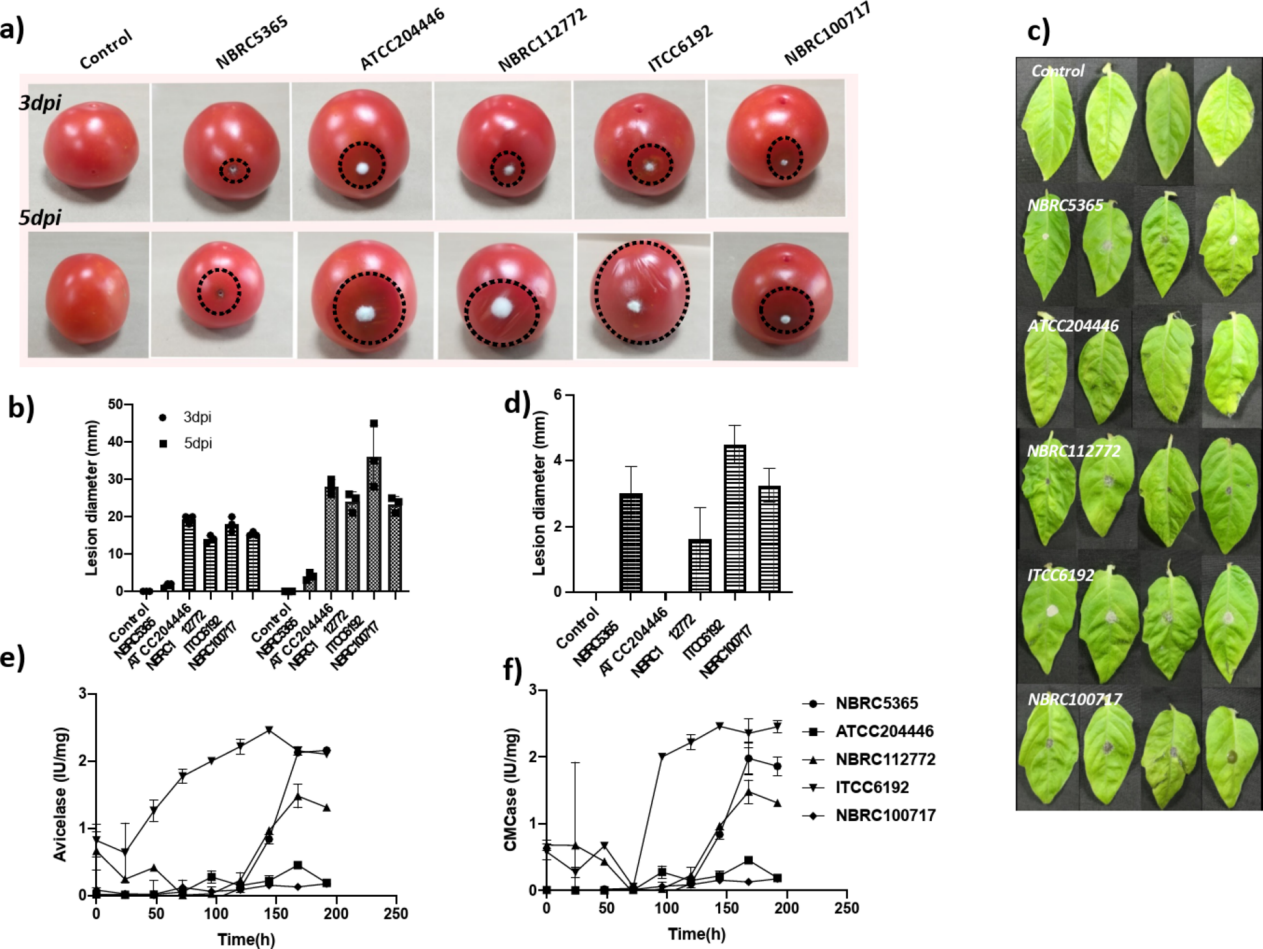
Selection of pathogenic Botrytis strain. Five strains of *Botrytis cinerea* were evaluated for pathogenicity using *in planta* and biochemical assay, (**a**) 1 × 10^6^ spores were inoculated in tomato fruit and infection was monitored after 3dpi and 5dpi. (**b**) Bar graph indicates lesion size in mm at 3dpi and 5dpi and the standard deviation was calculated from three independent infection experiments. (**c**) Leaf infection experiment was conducted by spotting 1 × 10^6^ spores on both the side of midrib and the image was taken 5 days post infection. (**d**) Bar graph indicates lesion size in mm at 5dpi and the standard deviation was calculated from four independent infection experiments. (**e**) Different Botrytis strains were grown in complex media and exo-glucanase activity was measured in the supernatant using avicelase assay. (**f**) Endoglucanase activity was measured in the culture supernatant based on CMCase assay. The experiment was done in triplicate and the standard deviation was calculated accordingly.

It was evident from *in planta* experiments that *Botrytis cinerea* NBRC5365 failed to colonize the fruit. In contrast, other strains displayed comparable infection at three days post-inoculation (dpi), with *Botrytis cinerea* ITCC 6192 showing maximum pathogenicity (Fig. 1a). The estimated lesion diameters also indicated the highest colonization for the ITCC 6192 strain, with lesion diameters of 20 mm and 35 mm at 3 and 5 dpi, respectively (Fig. 1b). Apropos to our findings on ripe fruit, ITCC6192 demonstrated the highest virulence in the detached leaf assay (Fig. 1c). This is clearly illustrated in the bar graph depicting highest lesion diameter in the leaf infected with ITCC6192 strain (Fig. 1d).

In vitro tests include estimating fungal growth and virulent enzyme secretion in response to plant biomass. For this, the Botrytis strains were cultivated in liquid culture with 2% plant biomass. The secretion of virulent proteins such as endo and exo-glucanases were estimated in the culture supernatant using avicelase and carboxymethyl cellulase (CMCase) tests. While all fungal strains showed similar growth patterns (data not presented), *Botrytis cinerea* ITCC6192 displayed the highest levels of exo-glucanaseFig.  1e) and endoglucanase activityFig. 1f). Overall, the experiments demonstrated that *Botrytis cinerea* ITCC6192 exhibited the highest pathogenicity in tomato plants compared to other Botrytis strains. Therefore, it was chosen for further study.

### Optimization of agar medium for proteomics experiment

The scrutiny of plant-pathogen interactions using proteomics is complicated by the presence of the proteomes from two species. An earlier study has demonstrated that the proteomic analysis of *Botrytis *sp. grown on mock infection media closely resembles the proteomics observed in plant infection studies^28^. Similarly, an agar media with plant extract has been shown as a potential media for inducing secretion of virulence factors in *Xanthomonas *sp. ^24^. Nevertheless, both studies yielded a limited number of protein hits. In this context, we have optimized a solid agar medium that more accurately simulates plant conditions, aiming to enhance proteome data discovery.

We have selected the ITCC6192 strain for our optimization experiments. However, elicitors optimized in the study will be broadly applicable for performing proteomics in other phytopathogenic fungi. A previous study has demonstrated variations in proteomics across six different wild-type Botrytis strains when subjected to the same media conditions^27^. The study revealed a mere 20% variability, identifying 48 unique spots among 225 protein hits, with most virulence factors recognized across all the strains. This suggests that a similar set of elicitors induces the secretion of virulence factors and establishes infection in different fungal strains, ensuring the broader applicability of media conditions across other susceptible hosts. This will lead to the identification of novel virulence factors in diverse phytopathogenic fungi that can help combat the pathogen effectively.

We prepared different test agar plates with (1) Media A-No substrate, (2) Media B-20% tomato extract, (3) Media C-20% tomato extract and 2% deproteinized plant extract (DPE), (4) Media D- 2% DPE, (5) Media E-2% Avicel and 2%DPE. An equal spore count (1 × 10^6^ spores) of the ITCC6192 strain was inoculated separately in test agar plates, ripe tomato fruit, and detached leaf from tomato plant and incubated at 22 °C for 96 h. Post-incubation, total fungal protein was extracted using phenol/acetone method from all the samples. The analysis of protein hits and abundance in different media conditions was conducted using mass spectrometry. A significantly higher number of proteins were observed in all test agar plates compared to leaf and tomato protein extract.

**Fig. 2:**
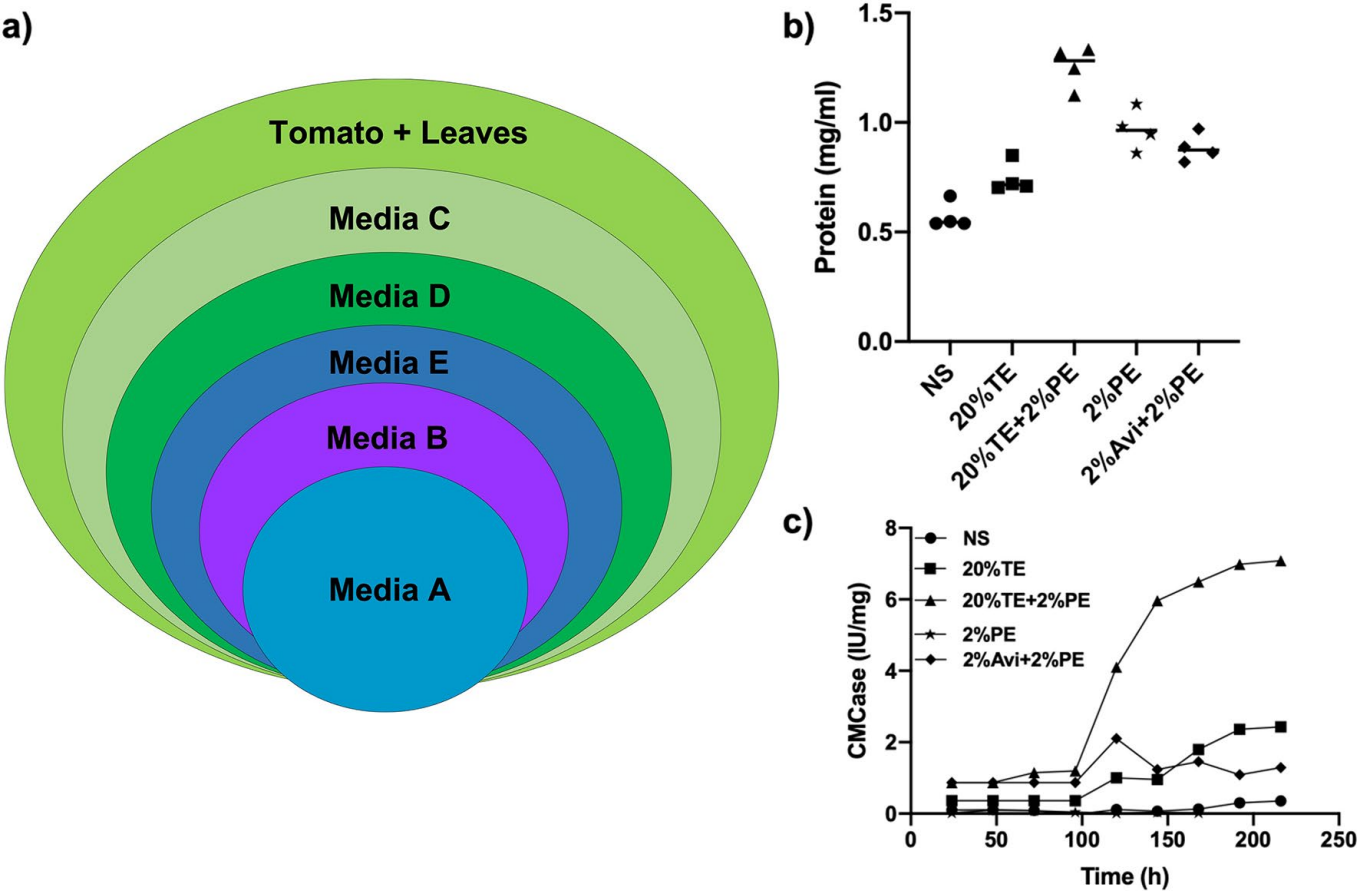
Optimization of media for proteomics. Media plates with differential composition of tomato extract and deproteinized cell wall were scrutinized to identify a suitable media, which closely mimics in plant condition, (**a**) 1 × 10^6^ spores inoculated in five different media with tomato leaf and fruit inoculation as control. The samples were subjected to MS analysis and overlap between different samples is represented in the form vein diagram. (**b**) *Botrytis cinerea *ITCC6192 was grown in defined media containing tomato extract (TE), or plant extract (PE) or, both. The supernatant was harvested after 96 h and analyzed for total protein concentration. c) Plant cell wall degrading activity (CMCase) was estimated in supernatant withdrawn every 24 h. The experiments were done in triplicate and standard deviation was calculated accordingly.

The analysis revealed that Media C with deproteinized plant extract and tomato extract showed the highest number of protein hits, with 474 proteins identified at a 1% FDR. In contrast, the protein extracts from the leaf and tomato displayed only 75 and 133 protein hits, respectively (Table S1). Additionally, the proteins found in Media C showed the highest correlation with those obtained from in vivo infection of tomato fruit and leaf samples (Fig. 2a). This underscores the importance of selecting the appropriate media for proteomic analysis.

In concordance with the above findings, the growth experiment also indicated that Media C promotes the enhanced secretion of total proteins (Fig. 2b). Additionally, the secretion of plant cell wall degrading enzymes (CMCase) was also overrepresented in Media C compared to other media conditions (Fig. 2c). In summary, the optimized media C, containing 20% tomato extract and 2% deproteinized plant extract, serves as an improved alternative for cultivating *Botrytis* sp. in proteomics research due to its enhanced protein discovery rate. This is the first instance where mock infection media has yielded protein hits from infecting fungi comparable to those observed *in planta* infections. As a result, we decided to use an agar medium with 20% tomato extract and 2% deproteinized plant cell wall for our temporal experiments.

### Stage-specific differential proteome expression in *Botrytis cinerea*

To gain insights into the regulation of pathogenesis in *Botrytis cinerea* and understand the early effector and late maintenance protein pool, protein isolates from different time points (12, 36, 72, and 120 h post-infection) were analyzed using label-free quantitative proteomics.

The proteomics data obtained were analyzed using iDEP2.01 online^29^. We quantified a total of 3244 proteins across all the time points at 1% FDR and 2045 fungal proteins were observed to be differentially regulated. This represents a very high number of Botrytis proteins quantified/identified than the previously reported for this pathogen grown under *in planta *conditions^19,24,30^. Our results are comparable to the only previous study^31^ where iTRAQ analysis yielded ~ 4000 hits, wherein the *Botrytis* sp. was cultivated on complex media rather than optimised minimal media.

**Fig. 3:**
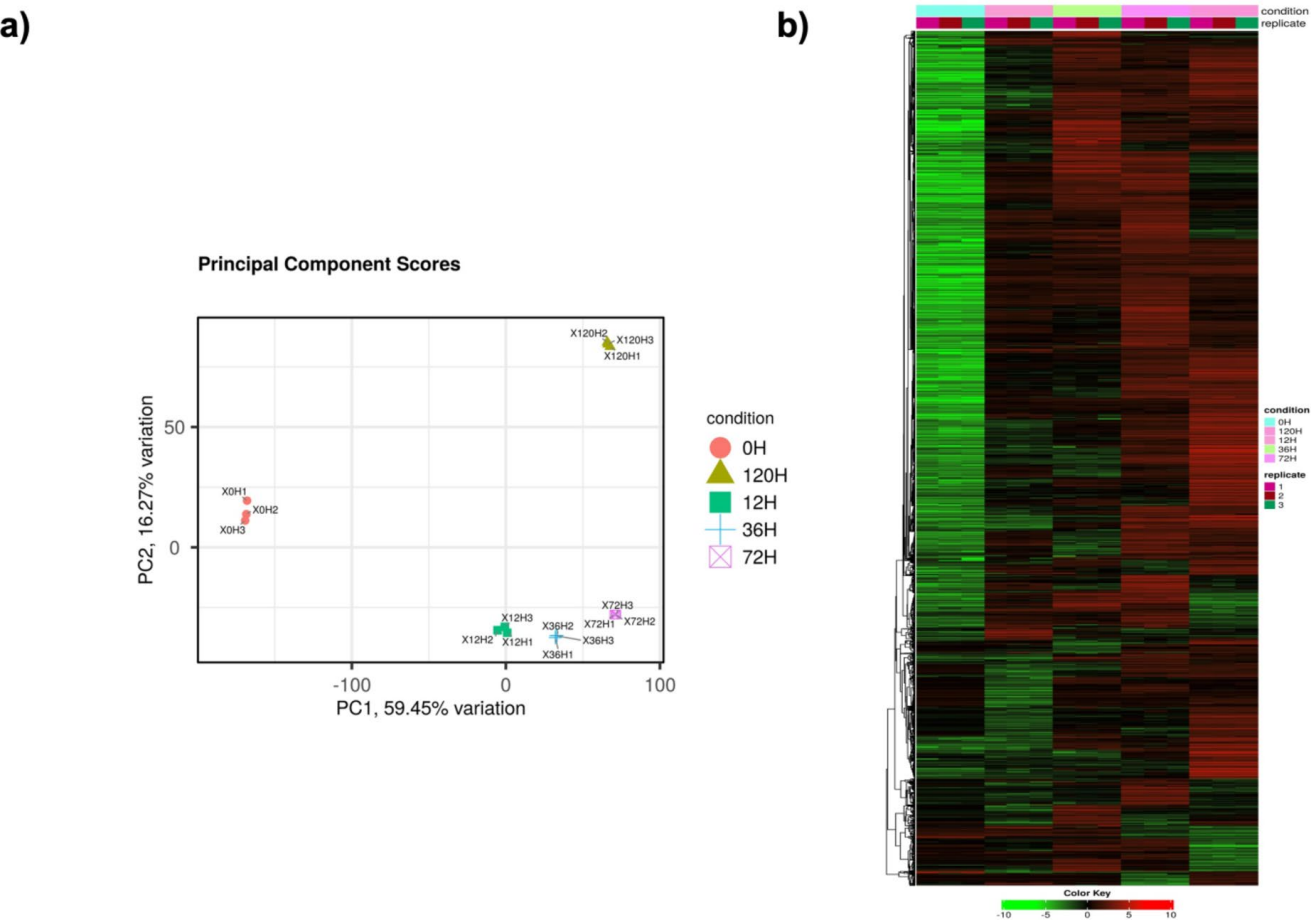
Lable-free quantitative proteomics at different time intervals. (**A**) Principal component analysis (PCA) of the proteomic samples at 0, 12, 36, 72 and 120 hpi. (**B**) Expression heat map of differentially expressed proteins at different phases of growth.

Principal component analysis (PCA) indicated a high degree of correlation among the replicates of each experimental condition (Fig. ​3a). The hierarchal clustering suggested differing patterns of protein expression in fungal tissue extracted at varying time intervals (Fig. 3b). This data concords with the literature suggesting the importance of protein dynamics in regulating growth phases in filamentous fungi^32^.

**Fig. 4:**
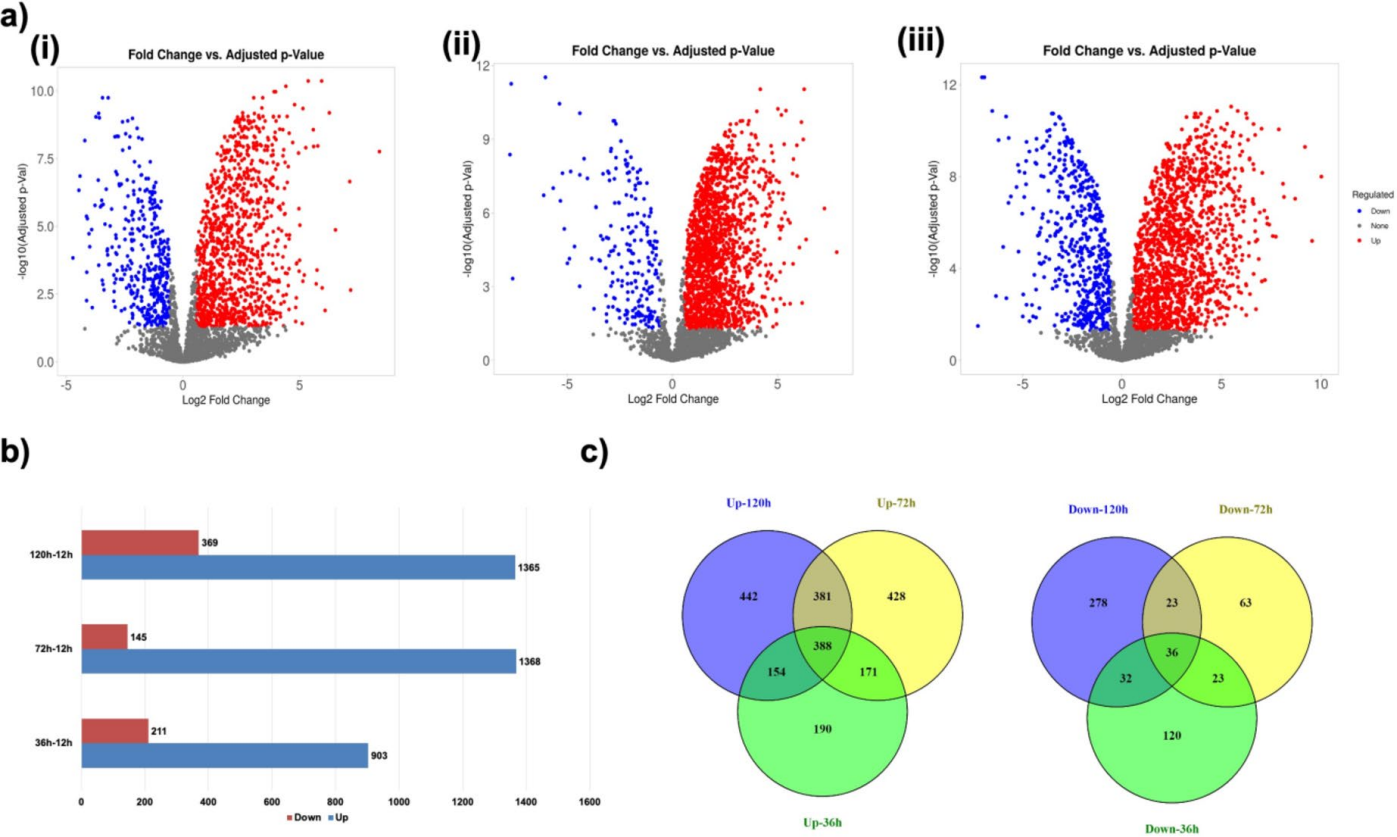
Identification and Label-Free Quantification of Proteins During Early and Late Growth Phase. (**a**) Volcano plots representing the results of the differentially expressed proteins from *Botrytis cinerea* at 36hpi (i), 72hpi (ii), 120hpi (iii) compared to 12 hpi. Highlighted (red) points represent proteins that showed higher abundance, whereas blue points represent downregulated proteins. In the volcano plot, the y-axis corresponds to the mean log10 expression value p-value), and the x-axis displays the log2 fold change value. (**b**) Bar graph indicating up and downregulated hits at various late growth phases. c) The Venn diagram shows consistent upregulation of 563 proteins and downregulation of 62 proteins in the late growth phase sample.

The volcano plot distribution of differentially expressed proteins at late phases of growth displayed a higher number of significantly up-regulated proteins compared to down-regulated ones (Fig. 4a). The results of the quantitative proteome analysis suggested that 903 proteins were upregulated at 36 h post-infection (hpi) compared to 12 hpi. This number increased to 1368 and 1365 proteins at 72 hpi and 120 hpi, respectively. On the other hand, 211, 145, and 369 fungal proteins were found to be downregulated at 36 hpi, 72 hpi, and 120 hpi, respectively, compared to 12 hpi (Fig. 4b). Additionally, the comparative analysis revealed that 388 proteins consistently showed upregulation throughout the infection, indicating their role in regular maintenance and cellular homeostasis (Fig. 4c). In contrast, only 36 proteins were consistently downregulated compared to 12 hpi across all the time points analyzed. As the fungus, *B. cinerea*, progressed from early (12 hpi) to later stages (120 hpi), its proteome profile witnessed substantial changes, with 278 unique proteins downregulated and 442 proteins upregulated only at 120 hpi.

Three categories of proteins including cell-wall degrading enzymes, proteases, catalase and proteins with unknown functions were observed as highly abundant during the early phase of infection, suggesting role of reactive oxygen species in penetration and establishment of infection during early plant-host interaction (Table. **S2**). This is in accordance with the previous study, which demonstrated that *B. cinerea *secretes free radical scavengers such as catalase and peroxidase to protect itself from the plant defence mechanism^33^.

**Fig. 5:**
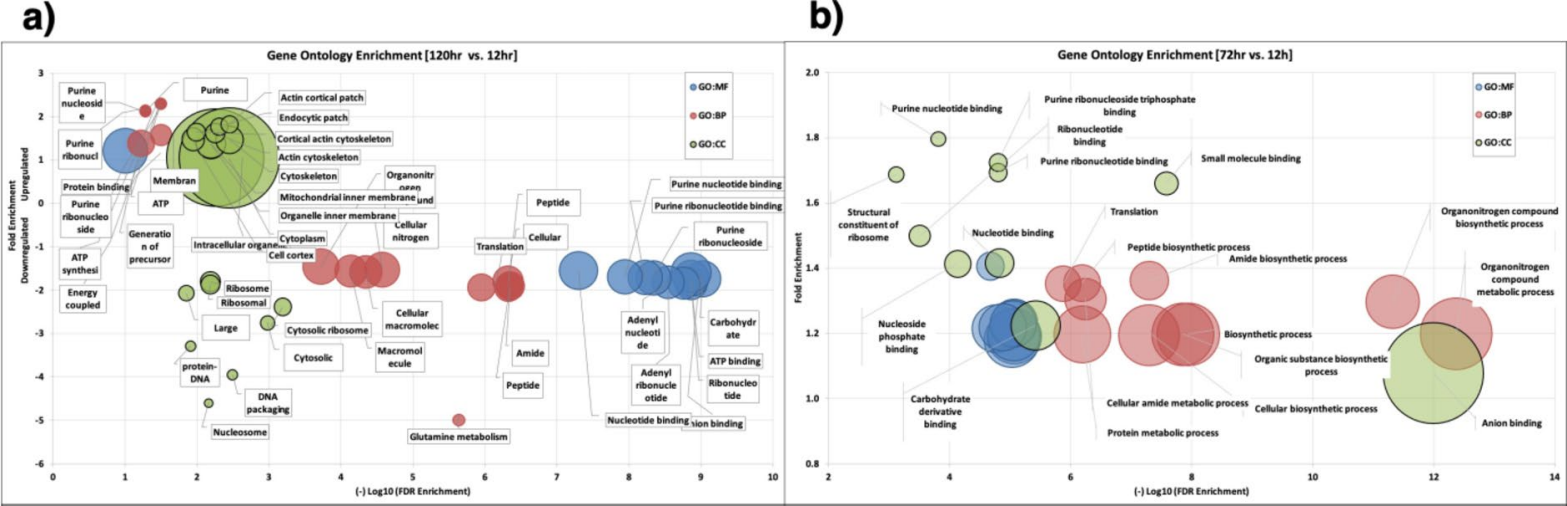
Gene Ontology enrichment analysis. (**a**) Gene Ontology enrichment analysis at 120 hpi, when compared with 12 hpi. (**b**) Gene Ontology enrichment analysis at 72 hpi, when compared with 12 hpi.

The expression trend analysis using K-means clustering dissected the differential expression pattern into 16 clusters (Fig. S1). Clusters 1, 2 and 11 indicate proteins that are highly expressed during the initial plant-host interaction such as glycosyl hydrolase, glycosyl transferase, protease, Rho GTPase, Rho GEF proteins. While cluster 2 and 4 predominantly represent proteins involved in the maintenance and survival of *Botrytis* sp. in the plant host such as BcRAC, Rac-like GTPase, golgi reassembly stacking protein, sorbitol dehydrogenase, endo-1,3-beta-glucosidase, peptidyl-prolyl cis-trans isomerase. These proteins were considered for gene ontology analysis (Fig. 5). Clusters 6, 9, 13, and 14 represent the proteins that showed homogenous expression throughout the growth stages analysed and constitute proteins involved in gene expression, cell survival, and metabolic processes (Table S3). This data demonstrates a strong correlation with the study which indicated the expression of virulence-related genes varies significantly across different stages of infection, species, and time points^34^.

The expression data showed a high correlation with the existing literature. Our data demonstrates the upregulation of the majority of plant elicitors, particularly RhoGEF, catalase, endopolygalacturonase, and endoglucanase, thus affirming the correctness and reliability of the data (Table 1). These early effectors play a crucial role in determining the outcome of plant-host interactions, either leading to infection or the expansion of lesions in the plant. Notably, the observed early activation of cell-wall modifying enzymes and hydrolases in fungi is responsible for breaching the physical barriers of the plant to support the intercellular proliferation of invading Botrytis hyphae. Additionally, the secretion of catalase and superoxide dismutase observed in the early phase proteome data serves as another effective strategy employed by phytopathogenic fungi to invade plant surface; this aligned well with Botrytis induced degradation of plant cell-wall observed in leaf detached assay (Fig. 1c). Overall, the proteins that are upregulated during the early phase contribute to the establishment of infection in plants.

**Table 1:** Correlation of proteins identified in Temporal proteomics with literature.

Gene/ GenBank ID	Function	Gene	Phenotypic effect of mutation	Probable Role	Reference
GenBank AAU87359 Lipase1	Penetration into the host surface	Lipase	Reduced infection with anti-lipase antibody	Responsible for early penetration (tomato)	Comménil et al. (1998)^35^
GenBank Z69264.1 Cutinase A	Penetration into the host surface	Cutinase	Decreased plant penetration in knockout	Involved in invasion(gerbera flowers )	Salinas et al. (1992)^36^
Gene ID 36,393,995 ***Bcspl1***	Plant defence	Ceratoplatanin	Overexpresed strain causes Increased Hypersensitive response to ROS	Responsible for increased resistance to plant defence (Arabidopsis)	Fr ´ıas et al. (2011)^37^
Gene ID 5,435,258 ***Bcpme1***	Plant cell wall degradation	Pectin methylesterase	Growth reduction on pectin medium observed in Bd90 mutant	Involved in colonization M (Apple fruits, grapevine and thale cress leaves) in Bd90 mutant only	Valette-Collet et al. (2003)^10^, Kars et al. (2005)^38^
Gene ID 5,430,570 ***Bcpg1***	Plant cell wall degradation	Endo-polygalacturonase	Growth reduction on pectin medium in Endo PG mutant	Involved in colonization (Apple fruits, tomato leaves and fruits)	ten Have et al. (1998)^17^
Gene ID 36,393,994 ***Bcxyn11A***	Plant cell wall degradation	Endo-β−1,4-xylanase	Delay in primary lesion formation, reduced colonization in knockout	Involved in colonization (Grape berries and tomato leaves)	Brito et al. (2006)^39^
GeneID 5,428,764 ***Bccat2***	Plant defence	Catalase	knockout showed increased accumulation of H2O2 in epidermal cells of the leaf sheath	Important for establishing early infection (rice)	Tanabe et al. (2011)^40^
GenBank KAK6606484.1	Plant defence	Peroxidase	knockout showed increased accumulation of H2O2 in epidermal cells of the leaf sheath	Important for establishing early infection (rice)	Tanabe et al. (2011)^40^
GenBank CAP12516.1 and CAP12517.1 ***BcnoxA*** and ***BcnoxB*** respectively	Plant pathogenesis	NADPH oxidase	bcnoxA and bcnoxB mutant showed attenuated virulence on citrus	Role in pathogenicity and formation of early lesion	Segmüller et al. (2008)^41^
Gene ID 5,440,666 ***Bctrr1***	Plant pathogenesis	Thioredoxin reductase	Knockout showed impaired virulence and more sensitive to oxidative stress.	Involved in virulence(Phaseolis vulgaris)	Viefhues et al. (2014)^42^
Gene ID 5,426,853 ***BcGlr1***	Plant pathogenesis	glutathione reductase	retarded infection as well as reduced growth on minimal medium	Important for germination(Phaseolis vulgaris)	Viefhues et al. (2014)^42^
Gene ID- 5,441,162 ***Bcsod1***	Plant pathogenesis	Cu-Zn‐superoxide dismutase gene	retarded development of lesions	Promote colonization (Bean leaves)	Rolke et al. (2004)^43^
GenBank CAD89674.1 ***BcGLOX***	Plant pathogenesis	glyoxal oxidase	mutants lost the ability to germinate and grow on minimal media	Critical for pathogenesis	Leuthner et al (2005)^44^

### Gene ontology and network analysis

The Gene Ontology (GO) analysis of these differentially expressed fungal proteins at different time points showed interesting results (Fig. 5a). Among differentially expressed proteins at 120 hpi, only one pathway, “protein binding,” was significantly enriched based on their molecular function. On the other hand, the down-regulated proteins enriched majorly into carbohydrate derivative binding, ATP binding, and ribonucleotide (adenylate/purine) binding categories, respectively. Based on biological processes, the fungal proteins involved in translation, peptide/nitrogen biosynthetic processes and overall macromolecule biosynthetic processes were downregulated at 120 hpi, which correlates with the GO enrichment results based on cellular compartment. The cytosolic and cytoskeletal proteins remain upregulated among other categories at later stages of the fungal life cycle (Fig. S2). At 72 h, the biological processes of translation, organonitrogen compound metabolic & biosynthetic processes, protein metabolic & peptide biosynthetic processes were enriched in the up-regulated fungal proteins, which is in total contrast with the proteins at 120 hpi and signifies the stage-wise progression of the fungal life cycle (Fig. 5b). Overall, the late growth phase demonstrated severe downregulation of genes involved in carbohydrate hydrolysis with consistent upregulation of maintenance protein, highlighting the metabolic pathways involved in initiating and establishing the infection in plants.

**Fig. 6:**
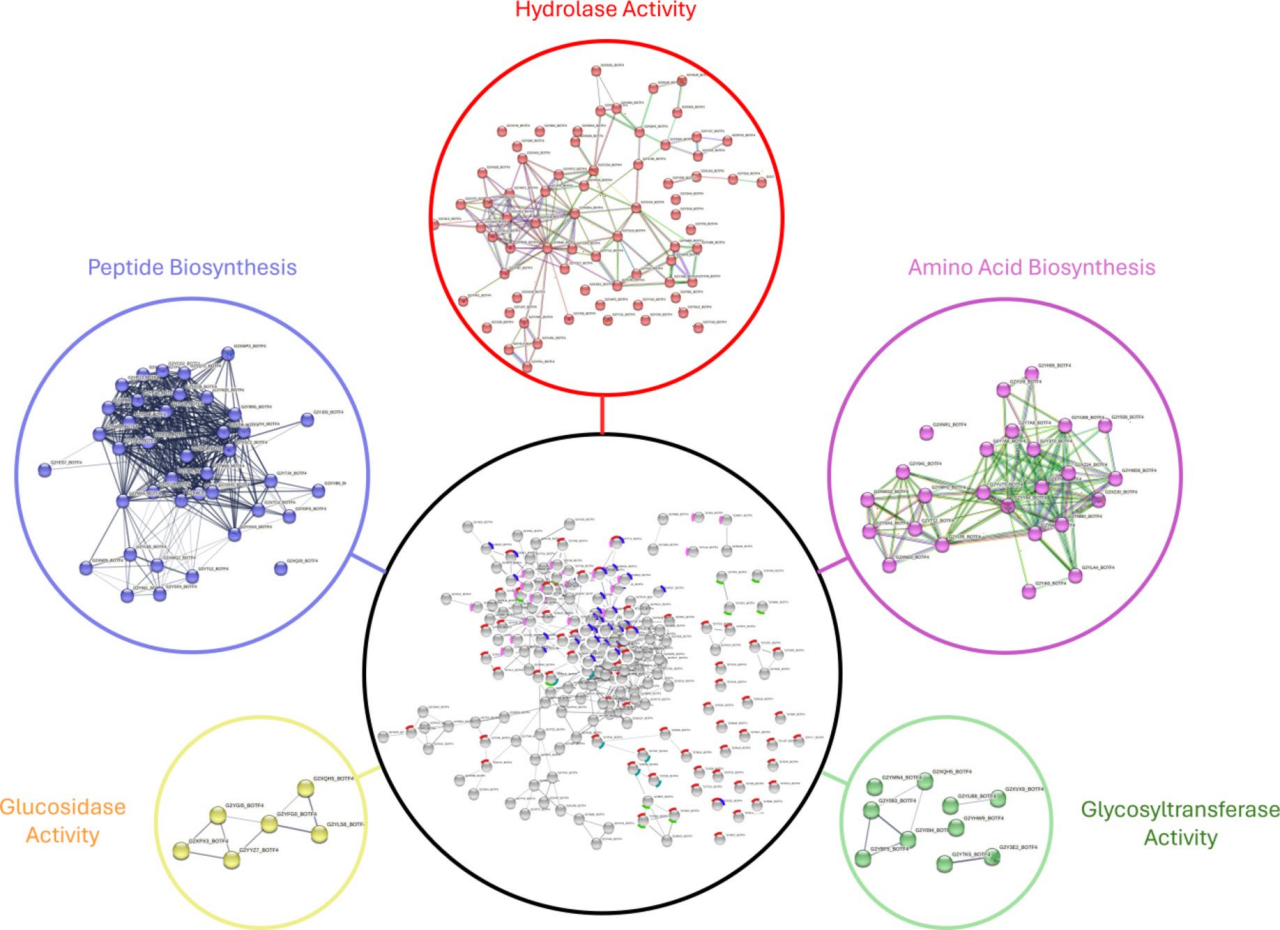
Protein-Protein Interaction (PPI) network analysis. Fungal proteins which showed lowered expression at late growth phases (72 and 120 hpi) as compared to early time points were subjected to interaction network analysis using STRING. The full network of these proteins is shown in the center, and the sub-networks of important categories of proteins are color-coded and represented in smaller circles.

After careful manual consideration, the fungal proteins that showed stage-wise differential expression showing down-regulation at late stages were selected and subjected to STRING analysis. This resulted in a network of 301 nodes (proteins) and 810 connecting edges (predicted associations) at high confidence for late growth phase. The important class enriched and exhibiting significant network connections include Hydrolases (71 proteins), especially glucosidases, hydrolyzing O-glycosyl compounds, suggesting that the fungus uses these hydrolases to penetrate the plant host (Fig. 6). This also ties in with the molecular function GO enrichment where in early-stage carbohydrate-binding activity related terms were over-represented during early growth phase. The other major sub-networks, including peptide and amino acid biosynthesis, are represented separately along with the full network (Fig. S3-S6).

The analysis of the proteome over time in *Botrytis cinerea* revealed that there were significant differences in how proteins behaved at different points. By categorizing the proteins that were regulated differently using gene ontology and KEGG pathway enrichment analysis, it was found that most of the effector proteins were present during the early stage of infection. This suggests that these effector proteins play a crucial role in the initial phases of the infection process. The study also allowed identification of several virulence factors such as cell-wall remodelling, catalase etc. However, the functional roles of these proteins still need investigation. Nevertheless, expansin, stress protein DDR48, phospholipase D, MyB domain containing protein, BOP1 (G-protein coupled receptor) and peptidase M20 are identified as the potential targets for further genetic analysis.

Overall, the study highlights the development and optimisation of a medium for proteomics research in phytopathogenic fungi. Our data aligned well with the previous literature on virulence factors while also identifying novel effectors such as Rho GTPase, Rho GEF proteins, BcRAC, Rac-like GTPase, Peptidyl-prolyl cis-trans isomerase. However, further studies are needed to identify the precise molecular functions, interactions, and regulatory pathways associated with these proteins. Understanding these effector proteins will help develop better strategies to combat fungus-mediated plant diseases effectively.

## Methods

### Strain and culture conditions

The five strains of *Botrytis cinerea*, ATCC204446 (ATCC, US), NBRC100717, NBRC112772, and NBRC536 (NBRC, Japan), and ITCC 6192 (ITCC India), were procured and maintained on potato dextrose agar (PDA) (Himedia, IN) at 22 °C. The spores were collected using extraction buffer (0.9% (w/v) NaCl and 0.1% (v/v) Tween 20). The spore count was estimated using a hemocytometer^45^.

For biochemical analysis, 1 × 10^7^ spores/ml were inoculated into the minimal media and incubated for 96 h at 200 rpm. The culture supernatant was collected every 24 h. For the proteomics study, 1 × 10^6^ spores were spotted on the minimal media-agar plates (0.08% glycerol, 0.2% bacto-peptone, 0.21% (NH_4_)_2_SO_4_, 0.3% urea, 0.03% MgCl_2_−7H_2_O, 0.03% CaC1_2_, and 0.1% metal solution (0.2%CaCl_2_, 0.5% FeSO_4_−7H_2_O, 0.15% MnSO_4_-H_2_O, and 0.17% ZnCl_2_in 28 mM HCl) in 100 mM potassium phosphate buffer (pH 6.0)) containing (1) 20% tomato extract; (2) 2% deproteinized plant extract; or (3) 20% tomato extract combined with 2% deproteinized plant extract, with no carbon serving as the negative control. The deproteinization of the plant tissue samples was done using the chloroform-methanol method^46^.

### Pathogenicity test

This experiment evaluated the pathogenicity of different *Botrytis *sp. on plants. For this, the tomato plant was grown under standard conditions, and the leaflets from a 3–4 week-old tomato plant (F1 hybrid) were used for the pathogenicity test. All the leaves and fruits were inoculated on the day of harvest to minimize variation^47^. Equal spores (1 × 10^7^) from *Botrytis *sp. were inoculated separately on the detached tomato leaves and prewounded fruits^48^. The infection was monitored regularly by measuring the diameter of lesion.

### Biochemical assays

The secretion of virulent enzymes such as cellulases was performed using a standard protocol to evaluate the plant cell wall degrading capability of different fungi. Briefly, 125 µl of CMC (2% w/v) in 50mM citrate buffer was mixed with 125 µl of appropriately diluted enzyme and incubated at 50 °C for 30 min; the reaction was stopped by the addition of 250 µl of Dinitro Salicylic Acid (DNS) and heating for 10 min at 100 °C. The amount of reducing sugar produced was estimated at 540 nm against glucose standards. The protein concentration was measured using the bicinchoninic acid assay (BCA) method, with bovine serum albumin (BSA) as the standard.

### Mass spectrometric analysis of fungal proteins

The fungal growth media being studied were assessed for their closeness to the plants using proteomic profiling. For this, an equal spore count (1 × 10^6^spores) of the ITCC6192 strain was inoculated separately onto test agar plates, ripe fruit, detached leaves and incubated at 22 °C for 96 h. The mycelia were then separated, and the total protein was extracted using the TCA/Acetone method. The total protein was quantified using BCA assay (Sigma, Germany) and subjected to in-gel trypsin digestion^27^ and subsequently analysed on LC-MS/MS for the identification of a total number of proteins in test conditions.

To evaluate the temporal progression of the fungal life cycle, the virulent Botrytis strain was grown on agar supplemented with 20% tomato extract and 2% deproteinized plant extract. 1 × 10^6^ spores were inoculated on the plate, and the fungal mycelia was harvested at different time points, such as 0, 12, 36, 72, and 120 h for proteome analysis.

Protein extraction from the samples was done using TCA-acetone method^27^. The extracted proteins were quantified using BCA assay (Sigma, Germany) and run on SDS-PAGE. The total protein extracts were subjected to trypsin digestion and subsequent LC-MS/MS analysis. A total of 50 µg of protein extract from each time point was first reduced with five mM TCEP and alkylated with 50 mM iodoacetamide and then digested with Trypsin (1:50) for 16 h at 37 °C. Digests were cleaned using a C18 silica cartridge and dried using a speed vac. The dried peptides were resuspended in buffer A (2% acetonitrile, 0.1% formic acid).

The peptide mixtures were subjected to label-free quantitative proteomics analysis on an Easy-nlc-1000 system coupled with an Orbitrap Exploris mass spectrometer. From each time point, 1 µg of peptide mixture was loaded on a 15 cm long C18 column, 3.0 μm Acclaim PepMap (Thermo Fisher Scientific) and separated with a 0–40% gradient of buffer B (80% acetonitrile, 0.1% formic acid*)* at a flow rate of 300 nl/min) over a period of 60 min. The MS1 spectra were acquired in the Orbitrap in profile peak mode at a resolution of 60000 in the mass range of 375–1500 Da with maximum injection time of 25ms and AGC target set to 300%. Dynamic exclusion was employed for 30s excluding all charge states for a given precursor. Top 12 precursors were subjected for MS2 fragmentation using HCD at a resoltuion of 15000 with maximum injection time of 22 ms and AGC target of 200%.

The raw files were analysed with Proteome Discoverer (v2.5) against the Uniprot *Botrytis cinerea* T4 database using the SEQUEST algorithm. The precursor and fragment mass tolerances were set at 10 ppm and 0.02 Da, respectively and the enzyme specificity was set for trypsin/P (cleavage at the C terminus of “K/R: unless followed by “P”). Carbamidomethyl on cysteine as fixed modification and oxidation of methionine was considered as variable modifications for database search. Both peptide spectrum match and protein false discovery rate were set to 1% FDR.

The lists of fungal proteins thus obtained were further analysed using iDEP2.01. to obtain meaningful outcomes and pathway enrichment of the data. The differential fungal proteome was thus obtained using DeSeq method and at 1% FDR. The missing values were compensated using group median. The GO enrichment analysis was also executed at 1% FDR. The selected and shortlisted differentially expressed proteins were subjected to interaction-network analysis using the STRING database, and the network thus obtained was visualized using Cytoscape.

## Electronic supplementary material

Below is the link to the electronic supplementary material.

Supplementary Material 1

Supplementary Material 2

Supplementary Material 3

Supplementary Material 4

## Data Availability

All data generated or analysed during this study are included in this published article and its supplementary information files. The entire analysed data is shared as a supplementary file with the manuscript, whereas the raw data has been submitted to the repository. The raw data is submitted to Indian Proteome Databank (IPD) at https://ibdc.dbtindia.gov.in/with project ID as IPD6285.
