# Supplementary material for: Temporal proteome profiling of Botrytis cinerea reveals proteins involved in plant invasion and survival
Source: Sci Rep. 2025 Apr 7;15:11857. doi: 10.1038/s41598-025-92683-5 (PMC11976908; doi:10.1038/s41598-025-92683-5)
Supplement: Supplementary file 4 — Supplementary Material 4 [file 41598_2025_92683_MOESM4_ESM.pdf]

**Supplementary Figures and Tables**

# Temporal proteome profiling of Botrytis cinerea reveals proteins involved in plant invasion and survival

Shriya Singh[1], Manasa Hegde[1], Inderjeet Kaur[2]* and Nidhi Adlakha[1]*

[1]Synthetic Biology and Bioprocessing group, Regional Centre for Biotechnology, NCR-Biotech Cluster, Faridabad, Haryana, India
[2]Department of Biotechnology, Central University of Haryana, Mahendergarh, Haryana, India

*Corresponding authors:
Nidhi Adlakha (ORCID:0000-0001-5153-1280): nidhi.adlakha@rcb.res.in
Inderjeet Kaur (orcid.org/0000-0001-8778-1396): inderjeet@cuh.ac.in

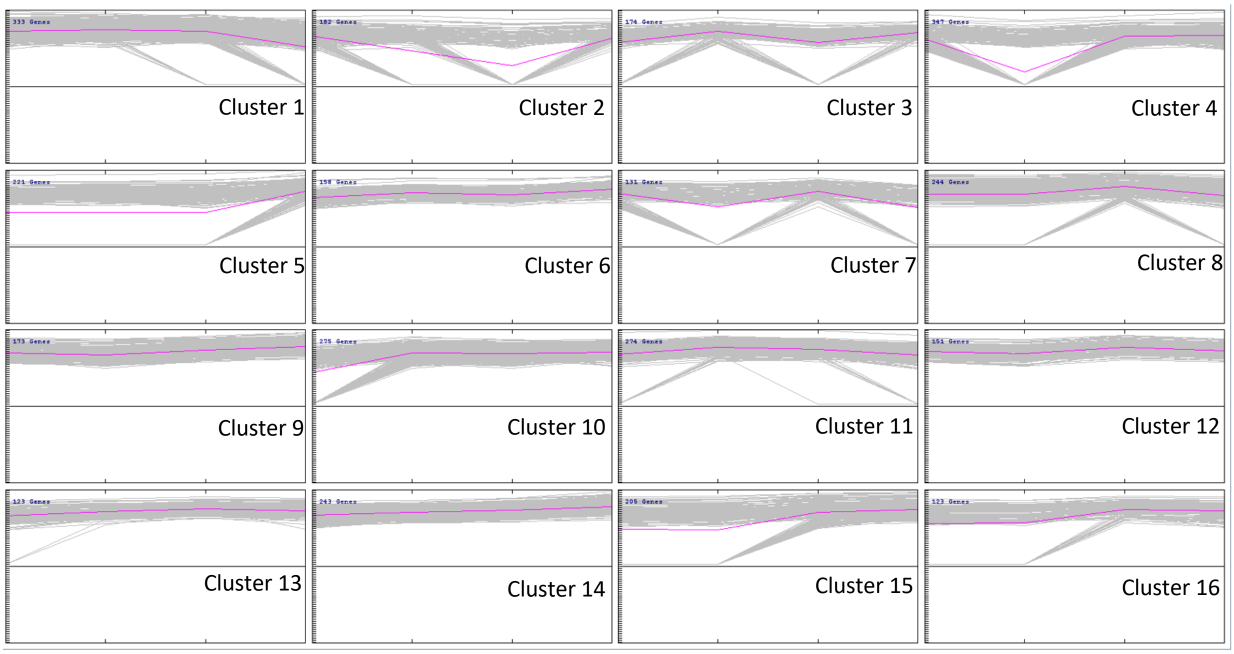


**Fig. S1 Expression trend analysis across different time points.** The open source platform MeV (Multiple Experiment Viewer) was used for the stage-wise expression trend analysis of the fungal proteins. K-means clustering analysis yielded 16 different clusters of the fungal proteins showing various expression trends over 120 hpi.

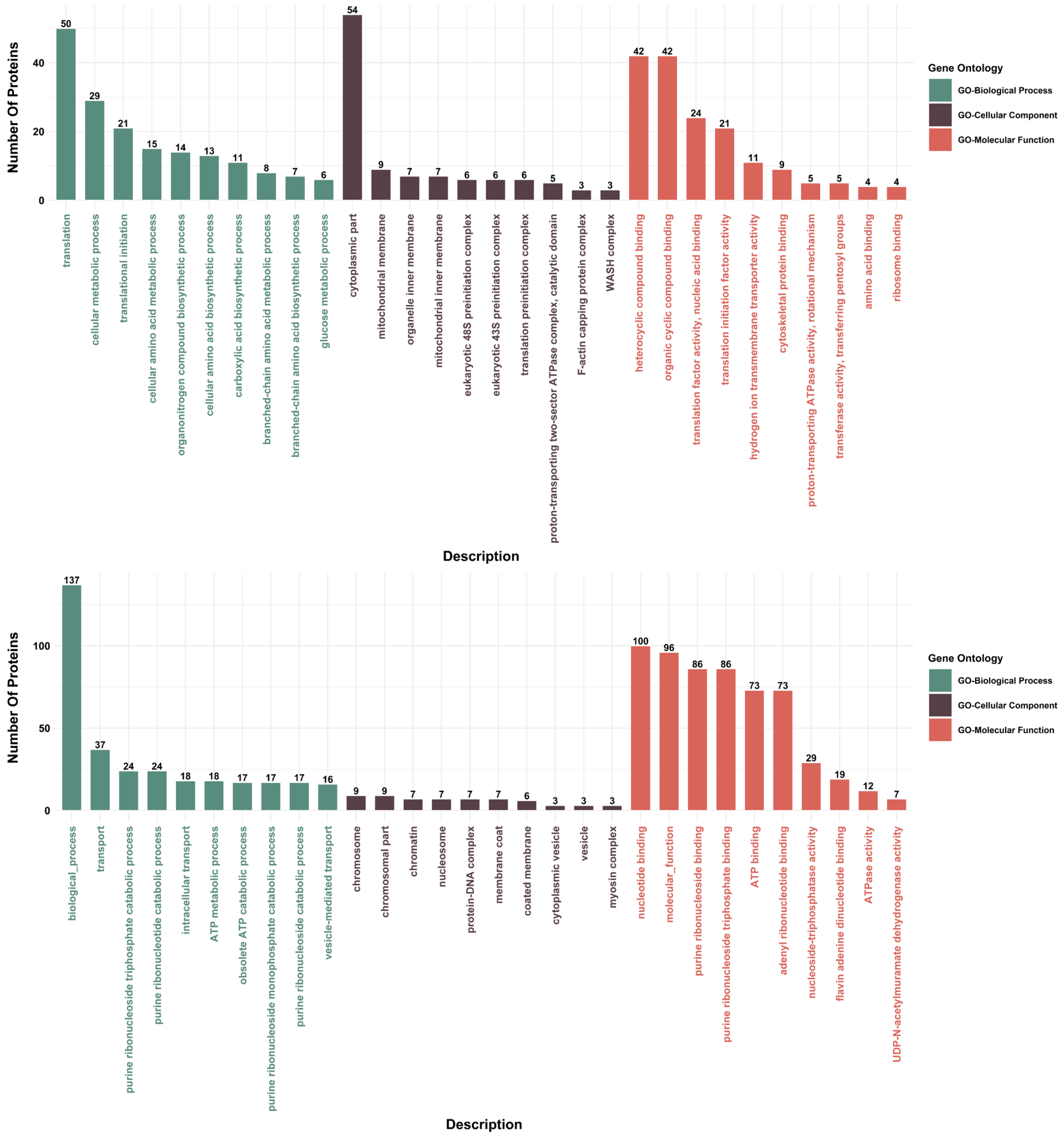


**Fig. S2 Gene ontology analysis** of top 10 upregulated (a) and downregulated (b) proteins at 120hpi

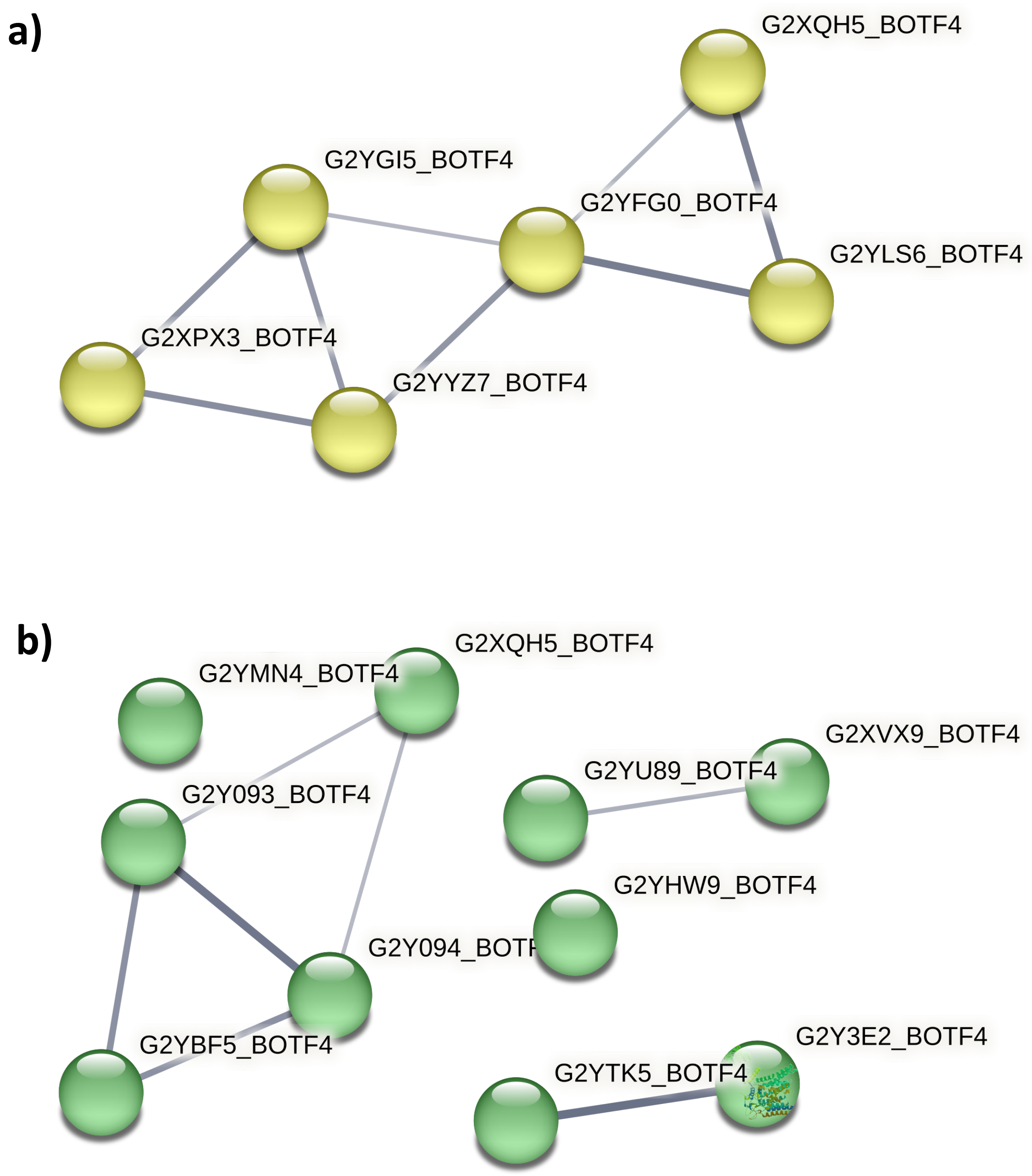


**Fig. S3** The protein-protein interaction network representing (A) Glycosidase activity and (B) glycosyltransferase activity

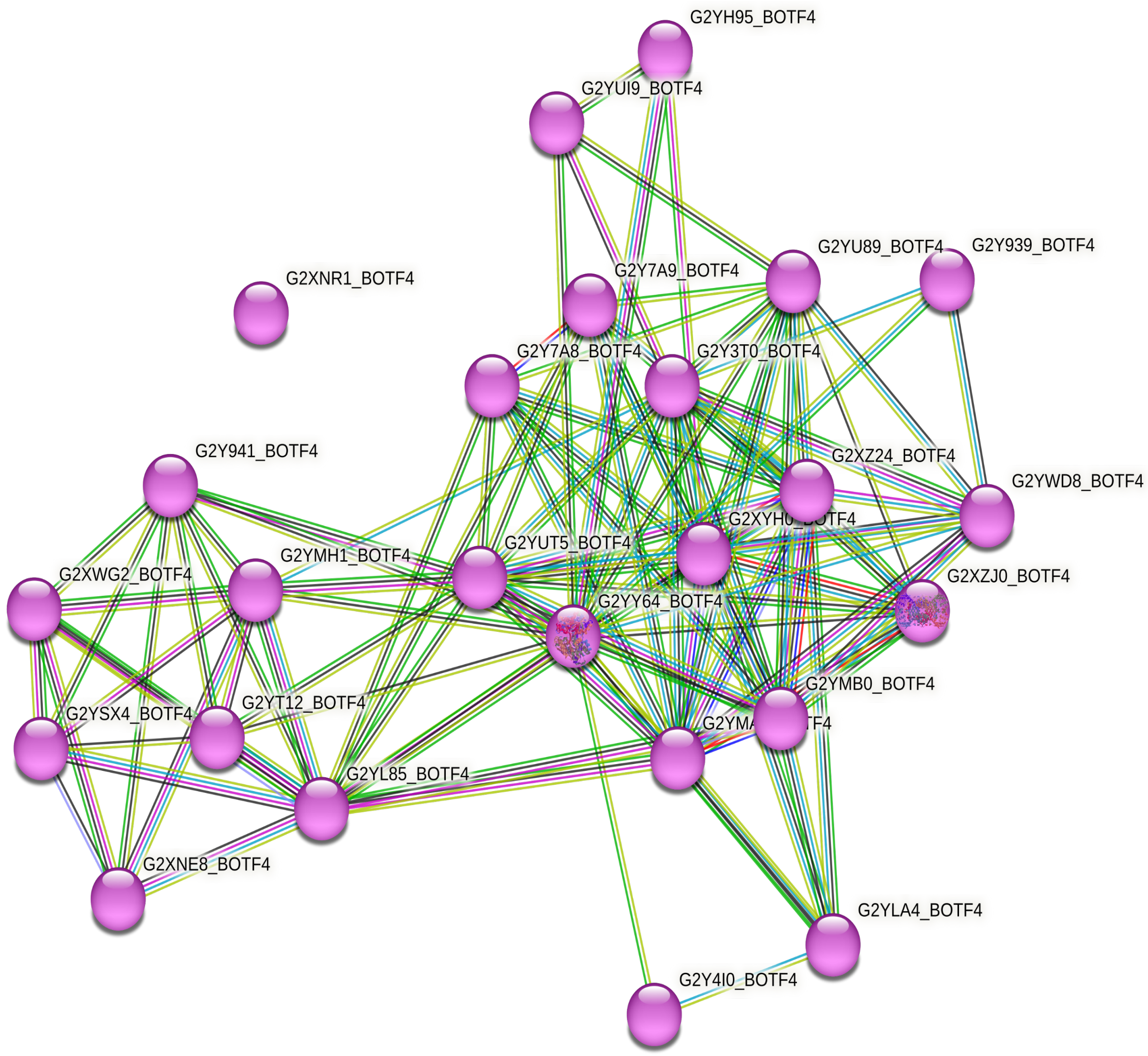


**Fig. S4** The protein-protein interaction network representing fungal proteins involved in amino acid biosynthesis

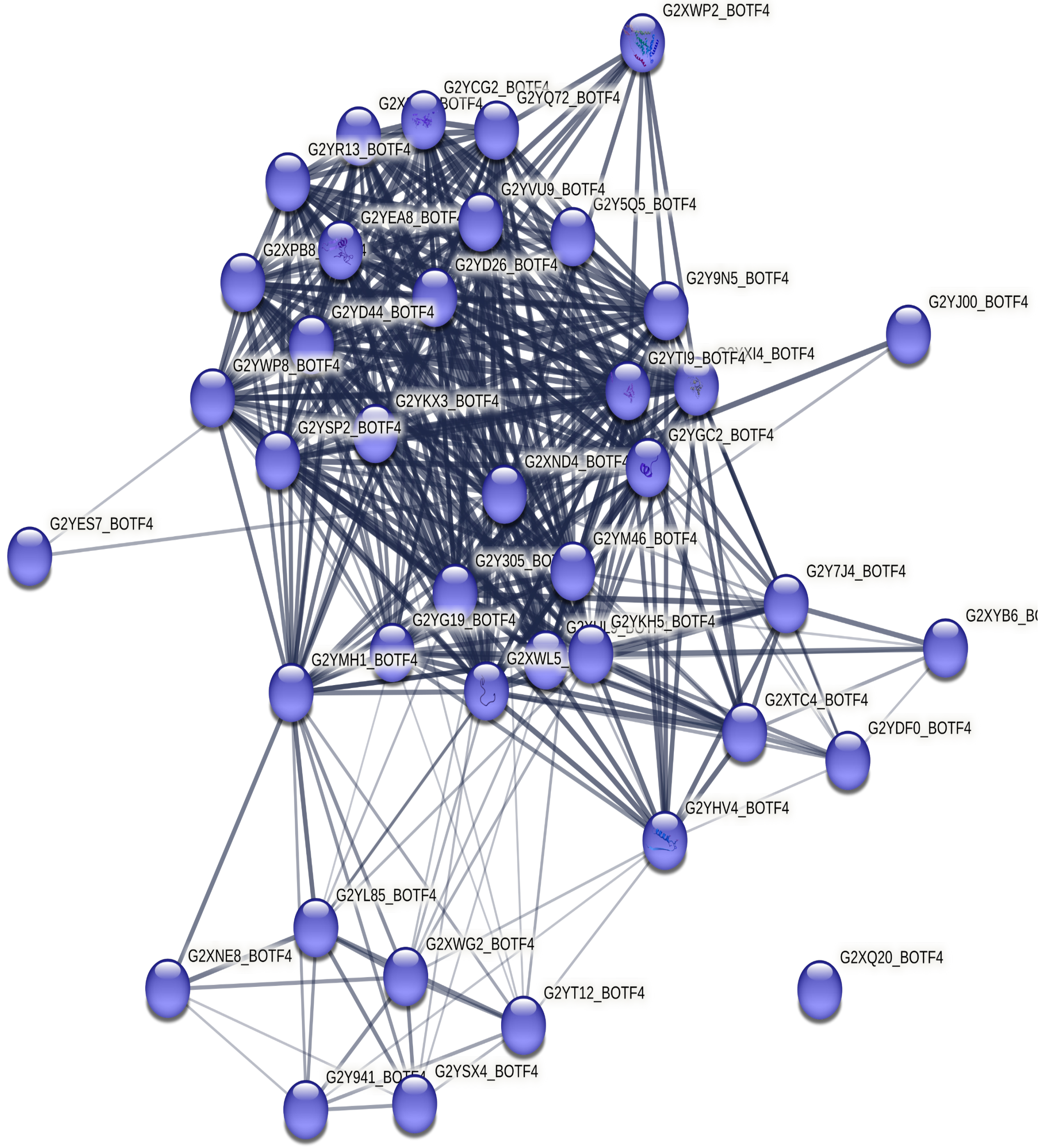


**Fig. S5** The protein-protein interaction network representing fungal proteins involved in peptide biosynthesis

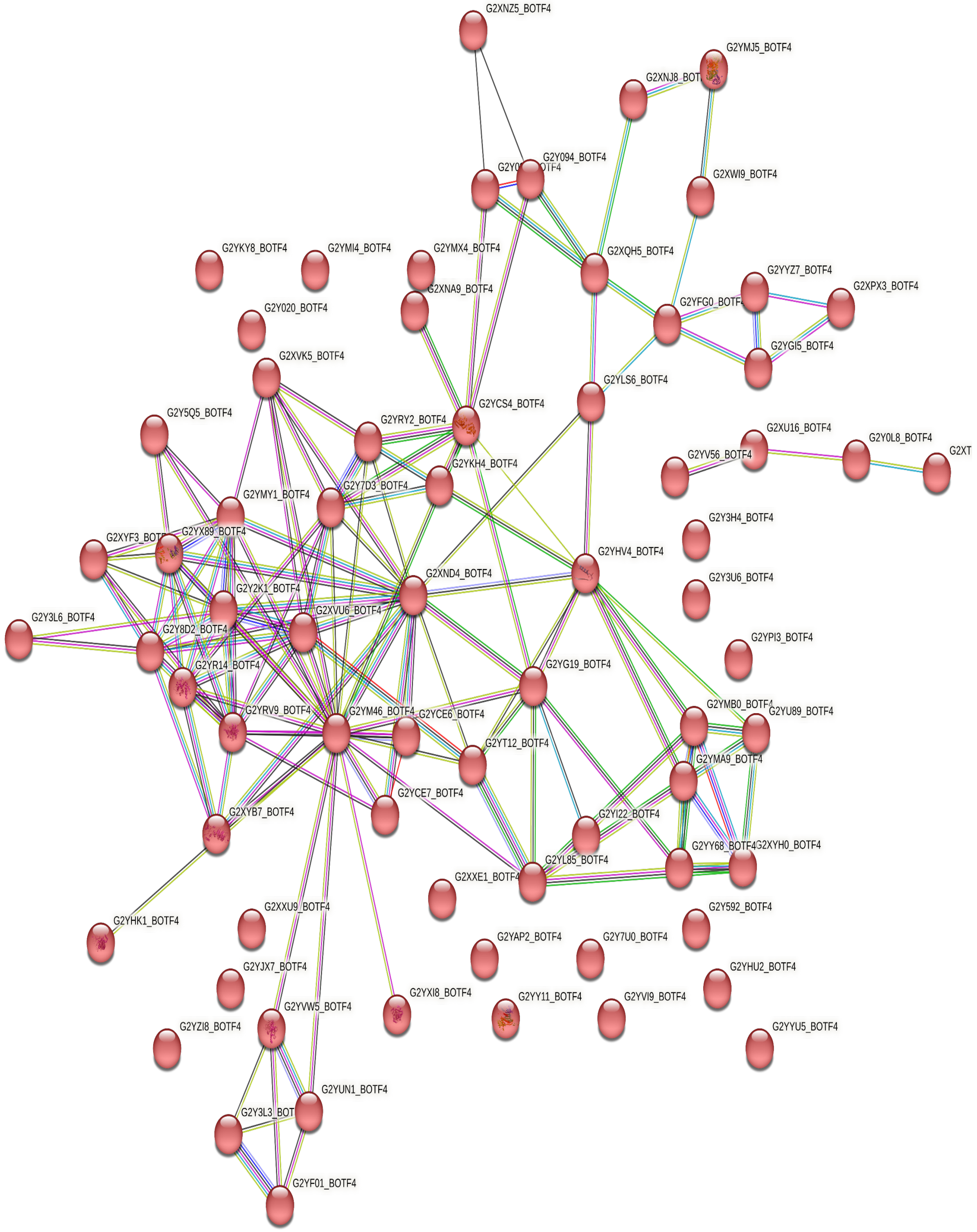


**Fig. S6** The protein-protein interaction network representing fungal proteins showing hydrolase activity

**Table S1: Differential Expression of proteins in synthetically designed media**

**Table S2: Temporal Expression of proteins in B. cinerea ITCC6192**

**Table S3: Cluster analysis of differential proteome data**
